# The association between initiation weekday of radiotherapy and local control in stage 1 glottic carcinoma: a retrospective analysis

**DOI:** 10.1093/jrr/rrae074

**Published:** 2024-09-17

**Authors:** Masashi Endo, Satoru Takahashi, Yukiko Fukuda, Kohei Okada, Kazunari Ogawa, Michiko Nakamura, Masahiro Kawahara, Keiko Akahane, Eri Murakami, Chiaki Shibayama, Ryutaro Onaga, Takafumi Nagatomo, Takeharu Kanazawa, Hiroshi Nishino, Harushi Mori, Katsuyuki Shirai

**Affiliations:** Department of Radiology, Jichi Medical University Hospital, 3311-1 Yakushiji, Shimotsuke, Tochigi 329-0498, Japan; Department of Radiology, Jichi Medical University Hospital, 3311-1 Yakushiji, Shimotsuke, Tochigi 329-0498, Japan; Department of Radiology, Jichi Medical University Hospital, 3311-1 Yakushiji, Shimotsuke, Tochigi 329-0498, Japan; Department of Radiology, Jichi Medical University Hospital, 3311-1 Yakushiji, Shimotsuke, Tochigi 329-0498, Japan; Department of Radiology, Jichi Medical University Hospital, 3311-1 Yakushiji, Shimotsuke, Tochigi 329-0498, Japan; Department of Radiology, Jichi Medical University Hospital, 3311-1 Yakushiji, Shimotsuke, Tochigi 329-0498, Japan; Department of Radiology, Jichi Medical University Saitama Medical Center, 1-847 Amanuma-cho, Omiya-ku, Saitama, Saitama 330-8503, Japan; Department of Radiology, Jichi Medical University Saitama Medical Center, 1-847 Amanuma-cho, Omiya-ku, Saitama, Saitama 330-8503, Japan; Department of Radiology, Jichi Medical University Hospital, 3311-1 Yakushiji, Shimotsuke, Tochigi 329-0498, Japan; Department of Radiology, NHO Tochigi Medical Center, 1-10-37 Naka-Tomatsuri, Utsunomiya, Tochigi 320-8580, Japan; Department of Radiology, Jichi Medical University Hospital, 3311-1 Yakushiji, Shimotsuke, Tochigi 329-0498, Japan; Department of Radiology, Saiseikai Utsunomiya Hospital, 911-1 Takebayashi-machi, Utsunomiya, Tochigi 321-0974, Japan; Department of Otolaryngology-Head and Neck Surgery, Jichi Medical University Hospital, 3311-1 Yakushiji, Shimotsuke, Tochigi 329-0498, Japan; Department of Head and Neck Medical Oncology, National Cancer Center Hospital East, 6-5-1 Kashiwa-no-ha, Kashiwa, Chiba 277-8577, Japan; Department of Otolaryngology-Head and Neck Surgery, Jichi Medical University Hospital, 3311-1 Yakushiji, Shimotsuke, Tochigi 329-0498, Japan; Department of Otolaryngology-Head and Neck Surgery, Jichi Medical University Hospital, 3311-1 Yakushiji, Shimotsuke, Tochigi 329-0498, Japan; Department of Otolaryngology-Head and Neck Surgery, Jichi Medical University Hospital, 3311-1 Yakushiji, Shimotsuke, Tochigi 329-0498, Japan; Department of Radiology, Jichi Medical University Hospital, 3311-1 Yakushiji, Shimotsuke, Tochigi 329-0498, Japan; Department of Radiology, Jichi Medical University Hospital, 3311-1 Yakushiji, Shimotsuke, Tochigi 329-0498, Japan; Department of Radiology, Jichi Medical University Saitama Medical Center, 1-847 Amanuma-cho, Omiya-ku, Saitama, Saitama 330-8503, Japan

**Keywords:** radiotherapy, glottic squamous cell carcinoma, local control, weekday

## Abstract

Radiotherapy is one of the definitive treatments for head and neck squamous cell carcinoma, especially early-stage glottic squamous cell carcinoma. Although there are several studies on the initiation weekday of cancer treatment, there are very few studies in the radiotherapy field. Thus, the present study investigated whether the initiation weekday of radiotherapy affects the local control rate for stage 1 glottic squamous cell carcinoma. A total of 105 patients with stage 1 glottic squamous cell carcinoma underwent definitive radiotherapy alone between 2007 and 2021. The group in which radiotherapy was started between Monday and Wednesday was compared with the group in which radiotherapy was started on Thursday or Friday. Sixty-seven patients started radiotherapy between Monday and Wednesday and 38 on Thursday or Friday. The 5-year local control rate was 98% (95% confidence interval: 94–100%) in the Monday–Wednesday group and 83% (95% confidence interval: 71–96%) in the Thursday–Friday group, with a significant difference (*P* = 0.005). On multivariate analysis including age, overall administration time (days), fractionation, irradiation field size and initiation weekday of radiotherapy, no factors other than initiation weekday affecting local control were identified. Radiotherapy toxicity did not differ between the two groups. For stage 1 glottic squamous cell carcinoma, starting radiotherapy on Thursday or Friday is associated with a lower local control rate; therefore, radiotherapy should be started by Wednesday.

## INTRODUCTION

Definitive radiotherapy (RT) is one of the standard treatments for head and neck squamous cell carcinoma (HNSCC). In early-stage glottic squamous cell carcinoma (GSCC), patients treated with RT alone have a good 5-year overall survival (OS) rate of 80.6–97.2% and a 5-year local control (LC) rate of 85.0–97.8% [1, 2]. Several factors have been reported to affect the outcome and are still being studied. In a related field, that of surgical oncology, treatment late in the week has been reported to worsen the prognosis of patients with cancer [3, 4] due, for example, to the accumulated fatigue of medical staff. In general, the intensity of work in RT is lower than in surgery, and the weekday effects caused by such factors are less likely to occur. However, to the best of our knowledge, there are few reports of weekday effects in RT. In the present study, patients with stage 1 GSCC who received RT alone at our institution were therefore retrospectively analyzed to determine the effect of the initiation weekday of RT. 

## MATERIALS AND METHODS

### Patients

A total of 106 patients with stage 1 GSCC who underwent definitive RT in our department between 2007 and 2021 were retrospectively reviewed. The patients had Eastern Cooperative Oncology Group performance status 0–1. The pre-treatment evaluations included physical examination, laryngoscopy, computed tomography and magnetic resonance imaging. The stage was determined according to the eighth edition of the Union for International Cancer Control. This study was conducted in accordance with the Declaration of Helsinki. The research protocol was approved by the Institutional Review Board of Jichi Medical University (22-068, 4 October 2022). To ensure that subjects had the opportunity to refuse to participate in the study, the study was posted on the website and at the facilities where the study was conducted (opt-out system) [5]. Written, informed consent was obtained from all patients in the pre-treatment period regarding the possibility of using their medical records for the study.

### Categorization

The patients were categorized for analysis into two groups: those in whom RT was started between Monday and Wednesday (Mon–Wed) and those in whom RT was started on Thursday or Friday (Thu–Fri). The initiation weekday of RT was left to the discretion of each radiation oncologist (authors). In no case was the clinician’s intention to initiate RT on a specific weekday recorded in the medical record, nor was the initiation of RT shifted to a specific weekday due to the date of admission. The patients received conventional RT with 2.0–2.4 Gy once-daily, 5 days a week, and accelerated hyperfractionation (AHF) with 2.0 Gy once on the first day and 1.4–1.5 Gy twice a day starting on the second day (the administration schedule is our empirical method), 5 days a week. The interfraction interval of AHF ranged from 4.5 to 6.0 hours. All patients in both groups were treated with 4- or 6-MV photons using opposing or oblique bilateral fields with the three-dimensional conformal RT technique. Acute and late adverse events were assessed in each group using the National Cancer Institute Common Toxicity Criteria for Adverse Events, version 5.0.

### Statistical analysis

The Kaplan–Meier method was used to analyze LC, laryngeal preservation (LP) and OS rates. LP was defined as a condition in which total laryngectomy was not performed and included conditions in which partial laryngectomy was performed. The prognostic relevance of variables was assessed using the log-rank test. A Cox proportional hazards model was created to examine factors associated with local recurrence. On multivariate analysis, explanatory variables included irradiation field size, overall administration time (OAT) and AHF or not, as per previous studies. Acute adverse events were compared between the two groups using Fisher’s exact test. A *P*-value <0.05 was accepted as significant. No sample size calculation was performed prior to the study. With a sample size of 67 cases in the Mon–Wed group and 38 cases in the Thu–Fri group, the post hoc power was calculated to be 0.49, assuming a 5-year LC rate of 95% in the Mon–Wed group and 85% in the Thu–Fri group and a significance level for a one-sided *P*-value <0.05. All data were analyzed using R statistical software (version 4.3.1) using Rstudio (RStudio PBC, Boston, MA, USA) with the aid of the libraries survival, survminer and gtsummary, and EZR (version 1.55, Jichi Medical University Saitama Medical Center, Saitama, Japan).

## RESULTS

Of the 106 reviewed patients, one was excluded from the analysis because of treatment with concurrent chemotherapy (docetaxel). The remaining 105 patients were analyzed. The median age of the patients was 71 years (range = 42–89 years). The median follow-up time from the initiation of RT was 57.4 months (range = 1.8–166.7 months). There were 67 cases in the Mon–Wed group and 38 cases in the Thu–Fri group, with 23 cases on Monday, 25 on Tuesday, 19 on Wednesday, 21 on Thursday and 17 on Friday. The median dose was 60.0 Gy (range = 60.0–66.8 Gy). The fractionation most performed was hypofractionation, using 2.2–2.4 Gy per fraction. The median OAT was 35 days (range = 28–51 days). The median irradiation field size was 6.0 cm (range = 5.0–7.3 cm) in width and 5.5 cm (range = 4.8–7.0 cm) in length. Table 1 shows the characteristics of the patients in the Mon–Wed and Thu–Fri groups.

**Table 1:** Patients’ characteristics

Factor	Overall	Mon–Wed	Thu–Fri
	*N* = 105^a^	*N* = 67^a^	*N* = 38^a^
Initiation weekday			
Monday	23 (22%)		
Tuesday	25 (24%)		
Wednesday	19 (18%)		
Thursday	21 (20%)		
Friday	17 (16%)		
Age (y)	71 (42–89)	71 (42–85)	72 (47–89)
Sex			
Female	4 (4%)	3 (4%)	1 (3%)
Male	101 (96%)	64 (96%)	37 (97%)
PS			
0	93 (89%)	58 (87%)	35 (92%)
1	12 (11%)	9 (13%)	3 (8%)
Total dose (Gy)	60.0 (60.0–66.8)	60.0 (60.0–66.8)	60.0 (60.0–66.0)
Fractionation			
Hypo	50 (48%)	33 (49%)	17 (45%)
AHF	44 (42%)	27 (40%)	17 (45%)
SF	8 (8%)	5 (7%)	3 (8%)
Hypo + AHF	2 (2%)	1 (1%)	1 (3%)
Hypo + SF	1 (1%)	1 (1%)	0 (0%)
Overall administration time (days)	35 (28–51)	35 (28–51)	35 (28–49)
Field size			
Length (cm)	5.5 (4.8–7.0)	5.4 (4.9–6.6)	5.8 (4.8–7.0)
Width (cm)	6.0 (5.0–7.3)	6.0 (5.0–7.0)	6.0 (5.0–7.3)

Mon–Wed: Monday to Wednesday, Thu–Fri: Thursday or Friday, PS: performance status, Hypo: hypofractionation, AHF: accelerated hyperfractionation, SF: standard fractionation.

^a^Median (range); *n* (%).

**Fig. 1:**
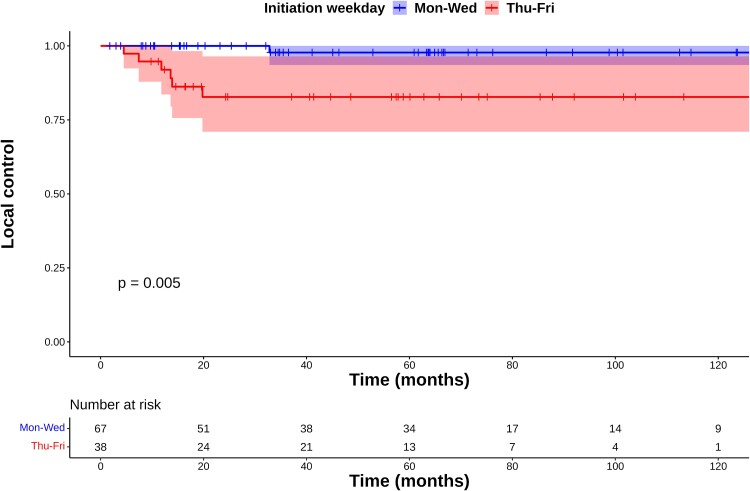
Kaplan–Meier plots of local control rates.

Of the 105 reviewed patients, 7 had local recurrences; all of the patients were male. One patient, four patients and two patients had started RT on Tuesday, Thursday and Friday, respectively. Two were treated with AHF and five with hypofractionation; none treated with standard fractionation had recurrences. The 5-year LC rate was 98% [95% confidence interval (CI): 94–100%] in the Mon–Wed group and 83% (95% CI: 71–96%) in the Thu–Fri group, with a significant difference (*P* = 0.005, Fig. 1).

**Table 2:** Univariate and multivariate analyses of local control

Factor	Univariate		Multivariate	
	*P*-value	HR (95% CI)	*P*-value	HR (95% CI)
Age (y)				
>71	0.79	0.815 (0.182–3.642)	0.46	0.546 (0.112–2.672)
Overall administration time (days)				
>35	0.38	1.939 (0.434–8.665)	0.4	1.997 (0.112–9.820)
Fractionation				
Non-AHF	0.4	2.004 (0.389–10.33)	0.8	1.261 (0.420–7.319)
Field size (cm)				
Length < 5.5	0.99	1.005 (0.225–4.496)	0.82	1.192 (0.256–5.543)
Width < 6.0	0.39	2.035 (0.395–10.49)	0.33	2.344 (0.427–13.08)
Initiation weekday				
Thu–Fri	**0.005**	11.011 (1.325–91.53)	**0.02**	12.059 (1.418–102.59)

HR: hazard ratio, AHF: accelerated hyperfractionation, Thu–Fri: Thursday or Friday.

**Fig. 2:**
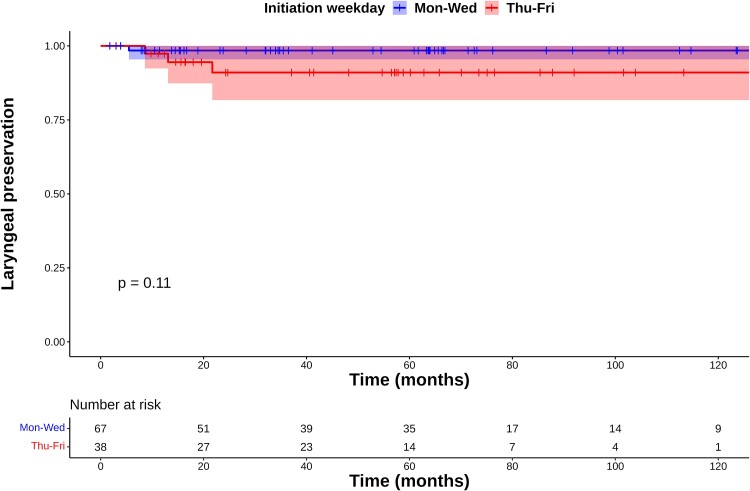
Kaplan–Meier plots of laryngeal preservation rates.

**Fig. 3:**
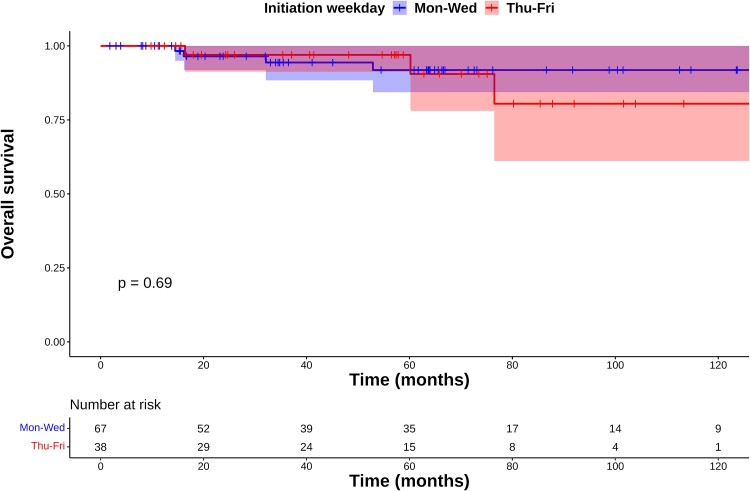
Kaplan–Meier plots of overall survival rates.

On multivariate analysis, only the starting weekday was a significant factor for LC (*P* = 0.02, Table 2). In the Mon–Wed group, one patient with local recurrence who started on Tuesday was controlled with partial laryngectomy, and the larynx was preserved. One patient who started on Monday developed Grade 4 laryngeal stenosis and required total laryngectomy. Of the 6 patients in the Thu–Fri group with local recurrence, 3 underwent partial laryngectomy, 3 underwent total laryngectomy and no patient required total laryngectomy due to adverse events. The 5-year LP rates in the Mon–Wed and Thu–Fri groups were 98% (95% CI: 96–100%) and 91% (95% CI: 82–100%), respectively, with no significant difference (*P* = 0.11, Fig. 2). Five patients died of other diseases, but none died of GSCC in the follow-up period. The 5-year OS rates in the Mon–Wed and Thu–Fri groups were 92% (95% CI: 84–100%) and 97% (95% CI: 91–100%), respectively (*P* = 0.69, Fig. 3). Table 3 shows acute adverse events. Two patients treated with AHF developed Grade 3 acute mucositis, and three patients treated with AHF developed aspiration pneumonia. These acute adverse events were appropriately treated and did not remain after the RT period.

**Table 3:** Adverse effects

Factor	Overall	Mon–Wed	Thu–Fri	*P*-value^a^
	*N* = 105^b^	*N* = 67^b^	*N* = 38^b^	
Dermatitis				0.33
Grade 0	1 (1%)	0 (0%)	1 (3%)	
Grade 1	63 (60%)	39 (58%)	24 (63%)	
Grade 2	41 (39%)	28 (42%)	13 (34%)	
Mucositis				0.5
Grade 1	18 (17%)	10 (15%)	8 (21%)	
Grade 2	85 (81%)	55 (82%)	30 (79%)	
Grade 3	2 (2%)	2 (3%)	0 (0%)	
Aspiration pneumonia	3 (3%)	2 (3%)	1 (3%)	>0.99

Mon–Wed: Monday to Wednesday, Thu–Fri: Thursday or Friday.

^a^Fisher’s exact test.

^b^ *n* (%).

## DISCUSSION

To the best of our knowledge, this is the first study to examine whether the initiation weekday of RT alone affects outcomes. Deciding which weekday to start RT is a very simple factor, which we believe will help many clinicians. Factors associated with worsening LC of RT for early-stage GSCC have been reported, including OAT [6], standard fractionation [7], smaller field size [8] and high photon beam energy [9, 10].

No reports have examined the effect of the initiation weekday of RT alone. The present study showed that starting RT on Thu–Fri was associated with a lower LC rate for stage 1 GSCC. One possible interpretation of the present results can be attributed to a feature of SCC called repopulation, which has been recognized as a mechanism of resistance to RT [11]. This phenomenon is associated with a worsening LC rate due to longer OAT; Fowler and Lindstrom [12] reported a 3–25% decrease in the LC rate with a 1-week prolongation of OAT. In Japan, a 1-week prolongation of OAT for the New Year holidays in December and January and Golden Week holidays in April and May was reported to worsen the 5-year LC rate from 89% to 74% [6]. In addition, it was recently reported that prolongation of OAT due to the Spring Festival in China was associated with worse outcomes in chemoradiotherapy (CRT) for nasopharyngeal carcinoma [13]. As shown, prolongation of OAT has been widely studied and found to be associated with worse LC in HNSCC, but in the present study, there was no difference in OAT between the Mon–Wed and Thu–Fri groups, which was not identified as a factor associated with LC. Repopulation is thought to begin to appear after 4 weeks of RT, with an increase in the dose required for tumor control [14]. Based on this factor, the JCOG0701 trial [15], which evaluated the efficacy of altered fractionation using 2.4 Gy once-daily, 5 days a week, for early-stage GSCC, indicated in the protocol that RT should be initiated by Wednesday (not mentioned in the article). In week 1 of the RT period, the number of days of irradiation was less for Thu–Fri initiation than for Mon–Wed initiation; the total dose at week 4 was therefore slightly lower. As an alternate hypothesis, although it is stated that repopulation occurs around week 4, further analysis in the report by Withers *et al.* [14] estimated that repopulation resistance is seen at least from day 25. Assuming this result is correct, if RT is given 5 times each week, the number of irradiations on day 25 of RT would be 19 on Monday, 19 on Tuesday, 18 on Wednesday, 17 on Thursday and 17 on Friday. This difference disappears on day 28 (20 times on all weekdays). These small differences might have caused the SCC to become resistant to RT, leading to worse LC. However, this was a single-center, retrospective study, which limited the findings that can be obtained, and further biological studies are needed.

A few reports have examined the impact of the initiation weekday of CRT on outcome. Mo *et al.* [16] analyzed the association between the weekday of initial RT induction and prognosis in 1440 patients undergoing CRT for nasopharyngeal carcinoma. They concluded that the initiation weekday of CRT did not affect the treatment outcomes of the patients. In addition to this report, Steber *et al.* [17] studied HNSCC of various primary sites, in which the initiation weekday of CRT also did not affect outcomes. Only these two reports have studied the association between the initiation weekday of CRT and treatment outcome. It has been suggested that chemotherapy has a mechanism to inhibit repopulation and reduce tumor resistance to RT [18]. This mechanism might be the reason why the outcome of CRT in both reports was unaffected by the weekday; however, as the report of the Spring Festival showed, prolongation of OAT might be associated with worse outcomes in CRT as well. There might be causes for the effect of the initiation weekday of RT on tumor control other than chemotherapy-induced inhibition of repopulation. Further studies are needed.

The present study had a few limitations. First, it was a retrospective study that included a small number of patients from a single center. The weekdays on which RT was initiated were therefore not random. Moreover, due to the small number of local recurrences compared to the number of analyzed cases, the factors reported in previous studies to worsen the LC rates were not identified as such in the present study, and the hazard ratios calculated in the multivariate analysis are probably uncertain. Second, AHF was indicated in a relatively large number of patients. In many cases, once-daily fractionation is probably indicated for early-stage GSCC. The results of the present study differ slightly from recent clinical situations. Third, the observation period was long. RT regimens for GSCC have changed significantly over the past few years, and the total dose and fractionation used in this study varied widely.

In conclusion, the initiation of RT alone on Thu–Fri was associated with a worse LC rate for stage 1 GSCC in the present study. If definitive RT alone is indicated, starting treatment on Mon–Wed should be considered, but further studies are needed to provide conclusive evidence of a causal relationship between the initiation weekday of RT and outcomes.

## References

[1] Chung SY, Kim KH, Keum KC et al. Radiotherapy versus cordectomy in the management of early glottic cancer. Cancer Res Treat 2018;50:156–63. 10.4143/crt.2016.503. 28301924 PMC5784634

[2] Nomura T, Ishikawa J, Ohki M et al. Multifactorial analysis of local control and survival in patients with early glottic cancer. Laryngoscope 2020;130:1701–6. 10.1002/lary.28240. 31397901

[3] Lagergren J, Mattsson F, Lagergren P. Weekday of esophageal cancer surgery and its relation to prognosis. Ann Surg 2016;263:1133–7. 10.1097/SLA.0000000000001324. 26565140

[4] Njølstad TS, Werner HM, Marcickiewicz J et al. Late-week surgical treatment of endometrial cancer is associated with worse long-term outcome: results from a prospective, multicenter study. PLoS One 2017;12:1–12. 10.1371/journal.pone.0182223. PMC5542466 28771617

[5] Eba J, Nakamura K. Overview of the ethical guidelines for medical and biological research involving human subjects in Japan. Jpn J Clin Oncol 2022;52:539–44. 10.1093/jjco/hyac034. 35349681 PMC9157286

[6] Nishimura Y, Nagata Y, Okajima K et al. Radiation therapy for T1,2 glottic carcinoma: impact of overall treatment time on local control. Radiother Oncol 1996;40:225–32. 10.1016/0167-8140(96)01796-3. 8940749

[7] Lyhne D, Douglas CM, Shaukat SI et al. Conventional fractionation should not be the standard of care for T2 glottic cancer. Radiat Oncol 2017;12:1–7. 29137654 10.1186/s13014-017-0915-8 PMC5686811

[8] Harwood AR, Hawkins NV, Rider WD et al. Radiotherapy of early glottic cancer. Int J Radiat Oncol 1979;5:473–6. 10.1016/0360-3016(79)90808-3 457491

[9] Izuno I, Sone S, Oguchi M et al. Treatment of early vocal cord carcinoma with 60CO gamma rays, 8/10 MV x-rays, or 4 MV x-rays-are the results different? Acta Oncol (Madr) 1990;29:637–9. 10.3109/02841869009090067. 2206580

[10] Parsons JT, Greene BD, Speer TW et al. Treatment of early and moderately advanced vocal cord carcinoma with 6-MV X-rays. Int J Radiat Oncol Biol Phys 2001;50:953–9. 10.1016/S0360-3016(01)01472-9. 11429223

[11] Schmidt-Ullrich RK, Contessa JN, Dent P et al. Molecular mechanisms of radiation-induced accelerated repopulation. Radiat Oncol Investig 1999;7:321–30. 10.1002/(SICI)1520-6823(1999)7:6<321::AID-ROI2>3.0.CO;2-Q. 10644055

[12] Fowler JF, Lindstrom MJ. Loss of local control with prolongation in radiotherapy. Int J Radiat Oncol Biol Phys 1992;23:457–67. 10.1016/0360-3016(92)90768-D. 1534082

[13] Xu C, Bin YK, Feng RJ et al. Radiotherapy interruption due to holidays adversely affects the survival of patients with nasopharyngeal carcinoma: a joint analysis based on large-scale retrospective data and clinical trials. Radiat Oncol 2022;17:1–11. 10.1186/s13014-022-02006-5. 35183221 PMC8858542

[14] Withers HR, Taylor JMG, Maciejewski B. The hazard of accelerated tumor clonogen repopulation during radiotherapy. Acta Oncol (Madr) 1988;27:131–46. 10.3109/02841868809090333. 3390344

[15] Kodaira T, Kagami Y, Shibata T et al. Results of a multi-institutional, randomized, non-inferiority, phase III trial of accelerated fractionation versus standard fractionation in radiation therapy for T1-2N0M0 glottic cancer: Japan Clinical Oncology Group Study (JCOG0701). Ann Oncol 2018;29:992–7. 10.1093/annonc/mdy036. 29401241

[16] Mo Y, Zhang B, Pan Y et al. Impact of the weekday of the first intensity-modulated radiotherapy treatment on the survival outcomes of patients with nasopharyngeal carcinoma: a multicenter cohort study. Oral Oncol 2021;116:105258. 10.1016/j.oraloncology.2021.105258. 33706048

[17] Steber CR, Russell GB, Rush MC et al. Timing of radiotherapy and chemotherapy start for patients treated with definitive concurrent chemoradiation for head and neck cancer. Acta Oncol (Madr) 2022;61:987–93. 10.1080/0284186X.2022.2086441. PMC9993330 35695175

[18] Kim JJ, Tannock IF. Repopulation of cancer cells during therapy: an important cause of treatment failure. Nat Rev Cancer 2005;5:516–25. 10.1038/nrc1650. 15965493

